# Electric Field-Induced Quenching of MAPbI_3_ Photoluminescence in PeLED Architecture

**DOI:** 10.1021/acsami.3c05880

**Published:** 2023-08-30

**Authors:** Rokas Gegevičius, Karim Elkhouly, Marius Franckevičius, Jevgenij Chmeliov, Iakov Goldberg, Robert Gehlhaar, Weiming Qiu, Jan Genoe, Paul Heremans, Vidmantas Gulbinas

**Affiliations:** † Department of Molecular Compound Physics, Center for Physical Sciences and Technology, Saulėtekio Avenue 3, LT-10257 Vilnius, Lithuania; ‡ Institute of Chemical Physics, Faculty of Physics, Vilnius University, Saulėtekio Avenue 9, LT-10222 Vilnius, Lithuania; § Department of Electrical Engineering, KU Leuven, Kasteelpark, Arenberg, 3001 Leuven, Belgium; ∥ IMEC, Kapeldreef 75, 3001 Leuven, Belgium

**Keywords:** perovskite, ions, photoluminescence, quenching, electric field

## Abstract

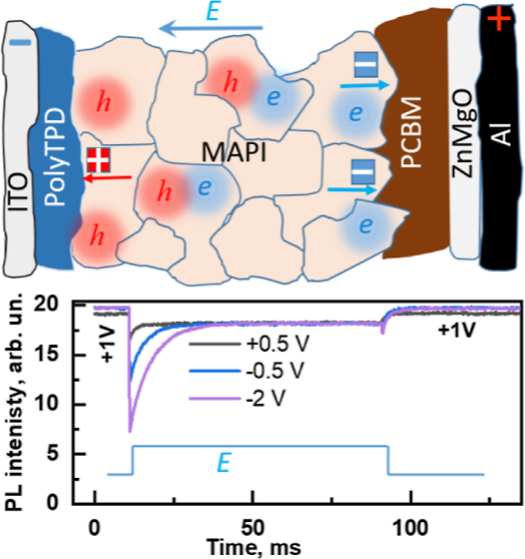

Photoluminescence (PL) measurements are a widely used technique for the investigation of perovskite-based materials and devices. Although electric field-induced PL quenching provides additional useful information, this phenomenon is quite complex and not yet clearly understood. Here, we address the PL quenching of methylammonium lead iodide (MAPbI_3_) perovskite in a light-emitting diode (PeLED) architecture. We distinguish two quenching mechanisms: (a) indirect quenching by slow irreversible or partially reversible material changes that occur gradually under the applied light and electric field and (b) direct quenching by the influence of the electric field on the charge carrier densities, their spatial distributions, and radiative recombination rates. Direct quenching, observed under the abrupt application of negative voltage, causes a decrease of the PL intensity. However, the PL intensity then partially recovers within tens of milliseconds as mobile ions screen the internal electric field. The screening time increases to hundreds of seconds at low temperatures, indicating activation energies for ion motion of about 80 meV. On the other hand, ultrafast time-resolved PL measurements revealed two main phases of direct quenching: an instantaneous reduction in the radiative carrier recombination rate, which we attribute to the electron and hole displacement within individual perovskite grains, followed by a second phase lasting hundreds of picoseconds, which is due to the charge carrier extraction and spatial separation of electron and hole “clouds” within the entire perovskite layer thickness.

## 1 Introduction

Photoluminescence (PL) measurements are one of the most important techniques for characterizing and studying perovskite materials. The PL quantum efficiency is directly related to the energy efficiency of perovskite light-emitting diodes (PeLEDs), while in the case of solar cells, the PL decay kinetics reveal information about the quality of the perovskite bulk and interfaces, as well as charge carrier extraction rates.^1,2^ The PL efficiency of perovskites and its dynamics depend on many material parameters, as well as their interfaces, the adjacent layers, temperature, applied and built-in electric fields, etc. Moreover, the PL efficiency also strongly depends on the perovskite treatment, storage, and investigation conditions. Therefore, formulating unambiguous conclusions solely on the basis of the PL data is often a difficult task.

A more advanced technique—electric field-induced luminescence quenching (EFILQ)—is another powerful tool to investigate the electronic processes in perovskites and their devices.^3^ Not only the externally applied electric field but also the built-in electric field present in solar cells or PeLEDs can be used for measurements since both can change the PL properties of perovskites. Nevertheless, the analysis of the PL quenching data and the obtained information is often complicated.

Several processes may be responsible for EFILQ and its observed signatures. In addition to the direct PL quenching caused by the influence of the applied electric field on the charge carrier recombination rate, the external voltage can change the charge carrier densities by carrier injection or extraction. Indirect processes, such as ionic motion, change of the trap population, modification of barriers for carrier extraction, etc., can also change the PL intensity under the applied voltage. Such processes have been widely discussed in the context of the hysteresis of perovskite solar cells (PSC),^4,5^ performance evolution of the PeLEDs under sustained voltage application, known as stressing,^6^ PL efficiency evolution under perovskite optical excitation, known as light soaking,^7–10^ observation of the moving dark front of the PL signal under voltage applied to perovskite films in lateral configuration.^11,12^ Most of these processes have been attributed to the dynamics of mobile ions and the chemical processes they trigger. In contrast, direct quenching of the perovskite PL has been less frequently addressed.

In this paper, we study EFILQ in MAPbI_3_-based PeLEDs operating in the reverse voltage regime. This type of device is very suitable for EFILQ investigation since no charge carriers are injected at reverse voltage. Moreover, in this regime, the equilibrium carriers are extracted, which reduces the electric field screening by the space charge that would otherwise be present. Based on our observations, we have identified three types of processes responsible for PL quenching: (a) direct influence of the electric field on the charge carrier densities and their distributions in the perovskite layer (electronic part), (b) electric field screening by mobile ions, and (c) slow irreversible or partially reversible changes related to the light soaking effect and the increase of *V*_oc_ analyzed elsewhere^13^ and active on second and minute time scales. We show that the slow changes are mainly responsible for the EFILQ at steady-state voltage, while the electronic part causes instantaneous PL intensity quenching under the application of negative voltage, which then partially recovers during tens of milliseconds at room temperature and during tens or even hundreds of seconds at low temperatures due to screening by mobile ions. Here, we focus on the electronic quenching part and how it is affected by the motion of ions (processes a and b). We distinguish two electronic quenching mechanisms attributed to the charge carrier separation within individual perovskite grains and within the entire perovskite layer thickness.

## 2 Results and Discussion

**Figure 1:**
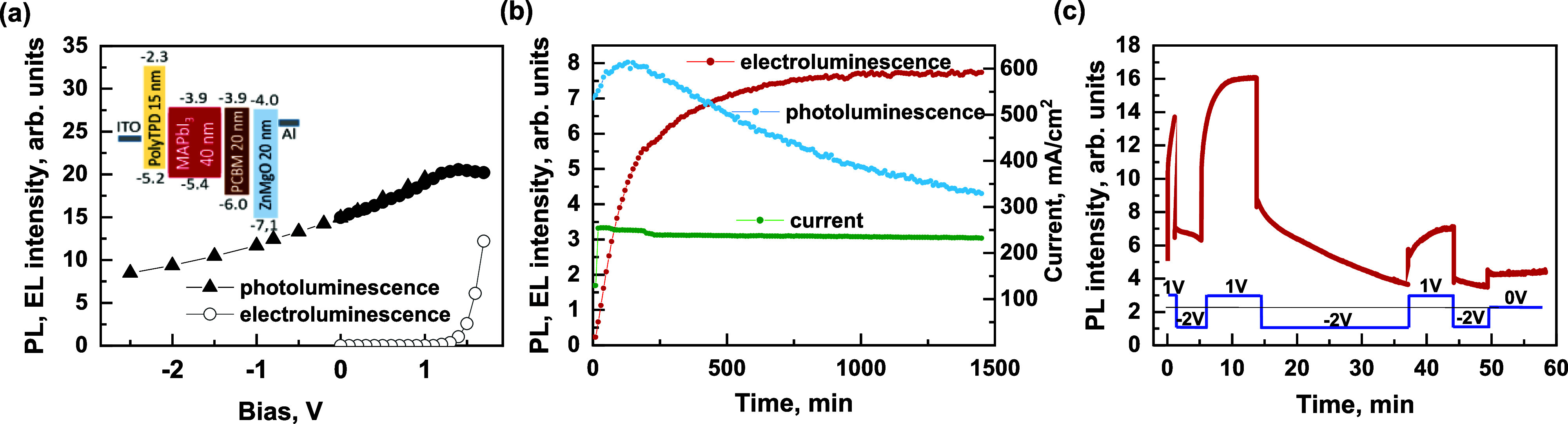
(a) Dependence of PL (filled triangles) and EL (open symbols) intensities of MAPbI_3_ PeLED on applied voltage. Inset in (a) shows the geometrical structure and scheme of electronic energy levels of the investigated PeLED. (b) Evolution of the EL (red) and PL (blue, left axis) intensities as well as electric current density (green, right axis) after the first-time application of the +1.5 V voltage; the PL intensity was measured by temporally (for 5 s) interrupting the applied voltage. (c) PL intensity measured by abruptly varying the applied voltage between −2 and +1 V, as shown in the inserted time chart.

During our measurements, we examined the EFILQ using several different voltage variations. In the simplest case, the PL intensity was measured under the sample excitation by a 635 nm, 5 mW CW laser by gradually changing the applied DC voltage between −2.5 and +1.7 V (Figure S4). The data obtained are presented in Figure 1a. For reverse voltages, the intensity of PL was integrated over 50 s for each voltage point (triangles). For forward voltages, the intensity of electroluminescence (EL) was measured without optical excitation, keeping the experimental conditions the same. Meanwhile, the PL signal for forward voltages was measured directly on top of the EL signal, and the real PL intensity was determined by subtracting the EL signal from the total luminescence intensity. It should be noted that this approach does not account for the possible interaction of injected and photogenerated charge carriers or the influence of photoexcitation on current. However, no significant changes in the evaluated PL signal when EL emerges and rises indicate that positive voltage above the EL threshold plays no exceptional role in PL modulation. Figure 1a shows that the PL intensity increases more than twice when the applied voltage is gradually increased from −2.5 to +1.2 V. At higher forward voltages (>+1.2 V), the PL intensity, which is obtained after subtracting the EL intensity, slightly decreased, mainly due to the sharp increase in EL signal that hinders the detection of the PL signal at higher voltages. The PL intensity is highest at +1.2 V, which approximately corresponds to the EL threshold voltage when carrier injection is still insignificant,^14^ but the internal electric field is approximately compensated by the external bias. The obtained voltage dependence of the PL intensity is also consistent with previous reports for PSC, where at short-circuit current conditions, the PL is strongly quenched as charge carriers are extracted before recombination. However, at open-circuit voltage conditions, PL quenching is minimized due to inefficient charge extraction^15,16^

Figure 1b shows the results of different types of measurements, in which both EL and PL intensities were recorded after the PeLED was first switched to the operating mode by applying a constant forward voltage of 1.5 V. The PL intensity in this case was measured by interrupting the applied voltage during the measurement time (5 s); thus, the PL measurements were always performed at 0 V. The EL intensity gradually increased on a time scale of minutes and hours, a process often referred to as sample stressing.^6^ The PL intensity also initially increased along with the EL intensity; however, after about 120 min, the PL intensity began to decrease while the EL intensity continued to increase. Since PL was always measured under the same conditions, these results show that slow irreversible or partially reversible material changes and not the electric field are responsible for the PL variations and that at least two types of different changes take place in the perovskite layer that cause both the initial increase and the subsequent decrease in PL intensity.

The picture described above becomes even more complex when the applied voltage changes abruptly. Figure 1c shows the PL dynamics induced by abrupt voltage changes between +1 V (not exceeding the PeLED operation threshold) and −2 V. The PL intensity changes in a very complex way, revealing a combination of instantaneous (within the time resolution of less than a second), hundreds of seconds, and even much slower processes. Moreover, the PL dynamics show that the PL intensity and its variations depend in a complex way on the history of previously applied voltages, which is consistent with the presence of several processes causing reversible and permanent changes of the device properties.^13^ However, we will not analyze the material modification processes in more detail here but concentrate on the electronic PL quenching part, which is responsible for the reversible fast PL changes.

**Figure 2:**
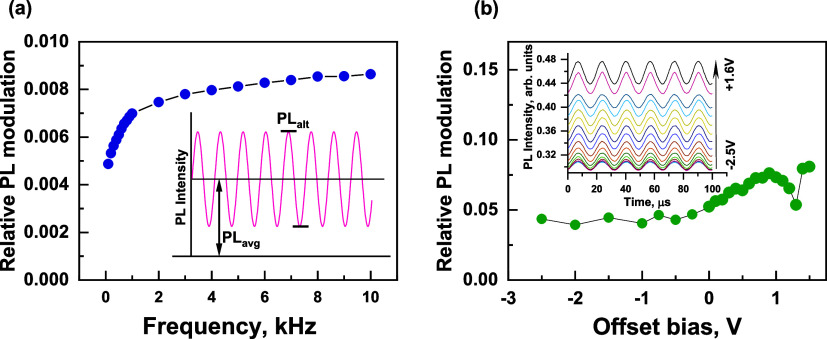
(a) Dependence of the relative PL modulation on the frequency of the sinusoidal 0.1 V voltage with a −1 V constant offset. The inset shows the average and alternating parts of modulated PL intensity. (b) Dependence of the relative PL modulation by the sinusoidal 6 kHz 0.1 V voltage on the constant voltage offset. The inset shows the dynamics of the modulated PL intensity at different offset voltages.

To better account for the fast PL modulation processes, we applied a sinusoidally modulated voltage of 0.1 V amplitude along with a constant offset. Figure 2a shows the PL modulation intensity divided by the average PL intensity as a function of voltage modulation frequency, which was varied in the range of 0.1–10 kHz. The intensity of PL modulation increases significantly with modulation frequency in the range of 0.1–1 kHz and stabilizes at about 9 kHz. Thus, this type of measurement is insensitive to the slow PL changes that occur on a time scale of seconds and indicates that the fast PL modulation occurs on a millisecond or even faster time scale. Since photochemical processes can hardly occur that fast, we attribute the fast PL quenching to the electronic processes that change charge carrier concentrations or their radiative recombination rate. The weaker PL quenching at lower frequencies suggests that some processes on a millisecond time scale weaken the PL quenching, most likely by reducing the electric field strength. Such processes are typically attributed to ion motion.^17–21^ We have also previously observed processes taking place in the identical PeLED on a millisecond time scale and attributed them to the motion of iodine ions.^17^

Figure 2b shows the dependence of the relative PL modulations PL_alt_/PL_avg_ (as shown in the inset of Figure 2a) induced by the 0.1 V AC voltage on the constant voltage offset. The measurements were performed at 6 kHz, when, according to Figure 2a, the electric field screening by the moving ions is already insignificant. The relative quenching is approximately constant at negative offset bias but increases almost twice at about +1 V and then decreases rapidly until it approaches the EL threshold voltage, at which ideally neither charge carrier extraction nor injection nor PL modulation takes place. A further increase of the offset voltage again results in a stronger PL modulation, which is caused by the emergence of the EL signal. However, the abrupt drop in modulation confirms the conclusion drawn earlier that the PL drop induced by an abrupt voltage change is actually caused by direct PL quenching by the electric field rather than by voltage-induced material modification.

**Figure 3:**
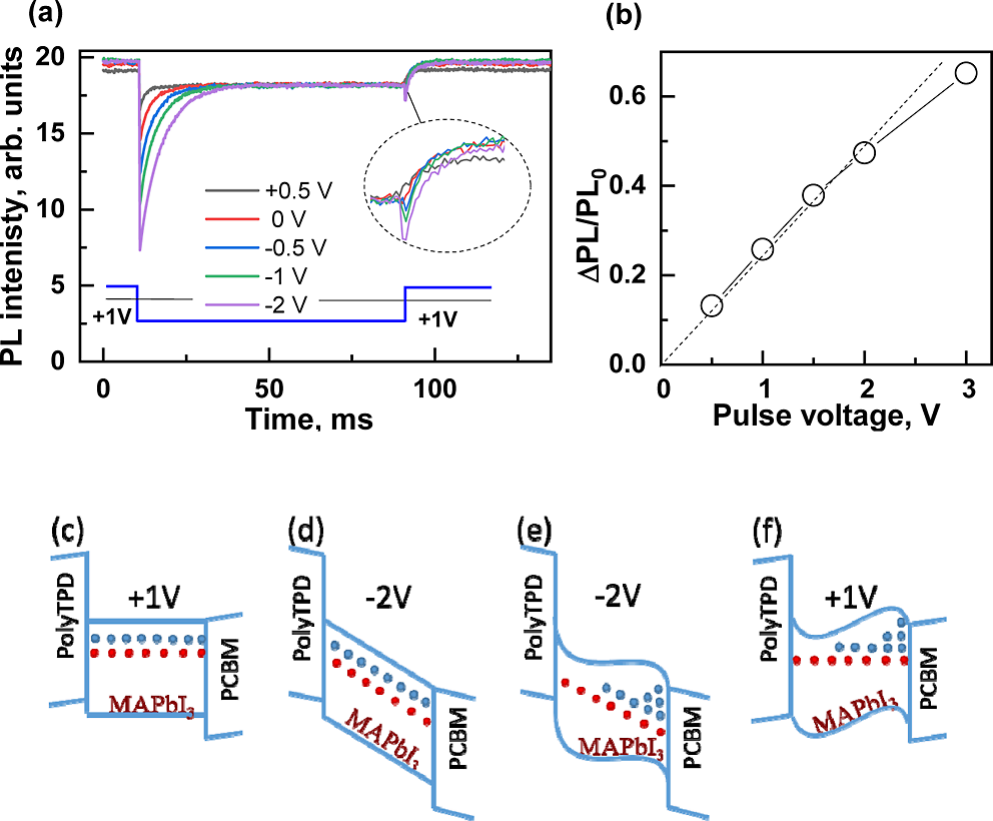
(a) PL dynamics induced by rectangular electrical pulses, measured at different negative pulse voltages with a constant +1 V offset. The inset shows the enlarged kinetics at the pulse termination time. (b) Dependence of the maximal PL quenching intensity on the voltage of the negative pulse. (c–e) Model of the electronic levels of the MAPbI_3_ PeLED: (c) under a steady-state compensating voltage of 1 V, (d) immediately after voltage drop to reverse voltage, (e) after electric field screening at reverse voltage, and (f) immediately after termination of the reverse voltage pulse. Blue and red dots indicate mobile (negative) and immobile/slow (positive) ions, respectively.

We further explored the EFILQ properties with a periodic rectangular modulated voltage. The periodic modulation allowed us to average the PL kinetics and track the PL dynamics with high time resolution. Figure 3a shows the PL dynamics when negative voltage pulses were applied relative to an offset. The measurement was performed by continuously applying rectangular negative voltage pulses for 80 ms, followed by a constant offset of +1 V for 120 ms, and tracking the PL intensity changes throughout the measurement period. The offset of +1 V was chosen to avoid PL quenching due to carrier extraction and to ensure that the internal electric field is weak or absent, referred to hereafter as a compensating voltage. The negative voltage drop resulted in an immediate (within ∼200 μs time resolution, RC of a sample of ∼0.9 μs) decrease in the PL intensity, consistent with effective PL modulation by high-frequency sinusoidal voltage. Figure 3b shows the voltage dependence of the initial relative PL quenching calculated as ΔPL/PL_0_ = (PL_0_ – PL_U_)/PL_0_, where PL_0_ is the PL intensity at a compensating voltage of 1 V, and PL_U_ is the PL intensity under the applied voltage pulse. The initial relative PL quenching was approximately proportional to the voltage drop and reached a slight saturation at large voltage drop values. The PL intensity was quenched more than twice at the highest voltage drop of −3 V used. The PL intensity partially recovered again during the following several or tens of milliseconds. We interpret this process to be a consequence of the spatial redistribution of mobile ions, which screen the external electric field.

Several unusual details of the PL dynamics deserve special attention. First, the recovery rate of PL intensity after application of a negative voltage pulse (Figure 3a) strongly depends on the pulse voltage. The partial PL recovery occurs with a time constant of about 1.6 ms after voltage drop from +1 to +0.5 V and with about 7 ms when the voltage drops to −2 V. Second, the PL intensity after its stabilization is remarkably independent of the voltage of negative pulses. Third, the PL intensity, as shown in the inset in Figure 3a, drops again for a short time when the negative voltage is turned off and then recovers quickly with similar dynamics as after the start of the negative pulse. Thus, the rapidly recovering PL drop occurs with both negative and positive voltage changes.

The model shown in Figure 3c–f explains these details. The applied voltage of +1 V approximately compensates for the built-in voltage (Figure 3c) and suppresses the EFILQ. The applied negative voltage initially creates an approximately homogeneous electric field distribution across the perovskite layer (the electric potential changes linearly, as shown in Figure 3d), causing strong PL quenching. The subsequent redistribution of ions almost completely screens the electric field within the large fraction of the perovskite layer, regardless of the applied voltage (Figure 3e), thus causing the partial recovery of PL to a level almost independent of the applied voltage. Moreover, screening of the weakly applied electric field requires only minor shifts of the ions, and this process is fast; it occurs within several milliseconds, as shown by the kinetics obtained at a low voltage pulse (Figure 3a, curve +0.5 V). On the other hand, the screening of the strong electric field requires the accumulation of a large fraction of the available ions, which drift over a large distance across the entire perovskite layer, so that the drift time increases to more than 20 ms at high voltages. PL quenching, induced by the termination of the negative voltage pulse, is also consistent with the proposed model. As shown in Figure 3f, immediately after the termination of voltage, the accumulated ions create an electric field of the opposite direction, which also causes a separation of the electron and hole distributions and consequently induces PL quenching, which gradually recovers as the accumulated ions diffuse. A redistribution of the internal electric field was also observed in mixed lead–tin perovskite systems.^18^

**Figure 4:**
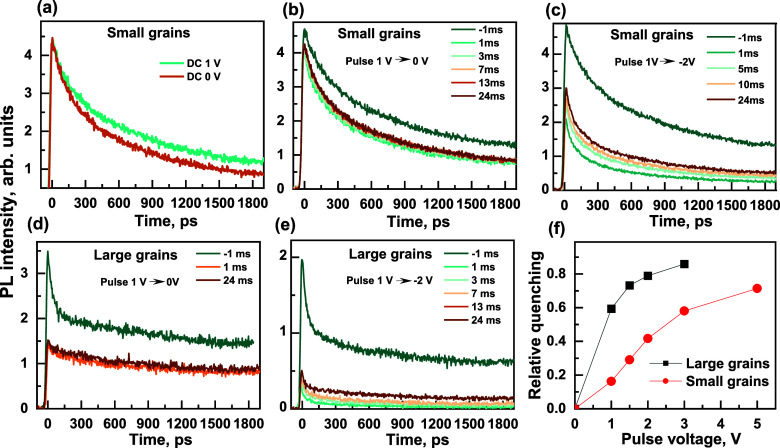
(a) Transient PL kinetics measured under +1 and 0 V steady-state voltages. (b–e) PL kinetics measured during the 30 ms-long negative pulse of −1 V (b,d) or −2 V (relative to the compensating +1 V voltage) (c,e) for small (upper plots) and large (lower plots) grain samples, respectively. The kinetics were measured at different delay times relative to the voltage pulse start. Kinetics at −1 ms were measured before the voltage pulse. (f) Relative PL quenching as functions of the pulse voltage for the samples with large and small grains.

To obtain additional details about the electronic quenching mechanism, we studied the ultrafast PL kinetics under the electric field generated by constant or pulsed voltages. We also measured the PL decay kinetics at different times after the application of the voltage pulse. Figure 4 shows the PL kinetics measured with a 100 nJ/cm^2^ excitation intensity under constant and pulsed applied voltages. PL decay at a constant compensating voltage of +1 V is slightly slower than at 0 V, which is consistent with the higher steady-state PL intensity (Figure 1a). Figure 4b,c shows the kinetics measured at different times before and after the application of rectangular negative pulses of −1 and −2 V, respectively, relative to the compensating 1 V voltage. As expected, the kinetics measured 1 ms before the negative pulse (labeled as “–1 ms”) were identical to those measured under a constant +1 V voltage. However, kinetics measured during the pulse action were sensitive to the timing of the optical pulse relative to the onset of the voltage pulse. Comparison of Figure 4a,b, also shows that the pulsed voltage causes more significant changes in PL kinetics than the constant voltage. Importantly, the initial PL amplitude was independent of the constant applied voltage (Figure 4a) but decreased when the pulsed voltage was applied. The difference is likely due to the different nature of the quenching mechanisms that occur at constant or pulsed voltages. The slow, irreversible or partially reversible material changes cause the appearance of additional PL quenching centers and thus change the PL decay rate. On the other hand, the fast electronic mechanism can reduce the charge carrier recombination rate very rapidly and thus affect the initial PL intensity as well. If we compare the PL kinetics before and immediately after the onset of the negative pulse, we find that the applied voltage causes an attenuation of the initial PL intensity and its faster decay during the first ∼500 ps. However, the PL decay rate in the 1000–2000 ps range remains remarkably independent of the applied voltage. The kinetics measured at longer times after the onset of the voltage pulse show both a weaker decrease in the initial PL intensity and a less significant acceleration in the PL decay rate, consistent with the electric field screening. Consequently, we can clearly distinguish between instantaneous and gradual PL quenching phases, often referred to as amplitude quenching and rate quenching, respectively. The rate quenching is consistent with the charge carrier extraction picture presented above. The field-induced acceleration of the PL decay rate is an expression of carrier extraction and redistribution of electron and hole “clouds”. Surprisingly, the electric field strongly accelerates the initial faster PL decay phase observed during the first hundreds of ps, but it does not change the second slower PL decay phase at times longer than 1 ns. The fast PL decay phase at zero electric field in MAPbI_3_ is usually attributed to the trapping of charge carriers.^1,19,20^ This attribution leads to the logical conclusion that the electric field strongly accelerates the extraction of free carriers but only weakly alters the kinetics of trapped carriers. On the other hand, the fast PL decay component has also been attributed to the redistribution of charge carriers, which occurs even when the internal electric field is zero.^21^ However, the detailed redistribution mechanism has not been discussed; therefore, it is difficult to speculate how this mechanism is consistent with the observed changes in the dynamics of PL under the electric field.

Special attention should be paid to the instantaneous PL quenching phase. It is much faster than the carrier extraction and spatial redistribution processes described above. Therefore, it is more likely due to the reduced radiative recombination rate of excitons or geminate charge pairs. The perovskite film of our studied PeLED consists of perovskite grains with a diameter of ∼10 nm. Under an applied electric field, the wave functions of electrons and holes within the single grain can be shifted in opposite directions, which decreases their radiative recombination rate. The carrier mobility within the single crystalline grain is expected to be much higher than the macroscopic mobility, which is reduced by the intergranular interfaces that form barriers for carrier motion.^22^ Therefore, the shift of the carrier wave functions within the single grain can be very fast, causing the instantaneous PL quenching phase.

To test this hypothesis, we examined EFILQ in a similar PeLED fabricated with larger grains (∼200–500 nm) similar to those used in high-efficiency solar cells. The different morphologies of small grain and large grain layers are observed through SEM images of the perovskite layers (Figure S2). Furthermore, the XRD analysis shows the main diffraction peaks to be located at 14.0 and 28.2°, which correspond to the (001) and (002) planes of the perovskite cubic phase. The full width at half-maximum (fwhm) of the large grain perovskite is ≈0.38°, compared to ≈0.46° for the small grain perovskite (Figure S3). As can be seen in Figure 4d,e, PL quenching was much more efficient in such a device. Figure 4f shows the dependence of the relative PL quenching on the pulse voltage for the samples with small and large grains. For small grains, the electric field can separate electrons and holes by only a few nanometers, i.e., the distance comparable to the exciton Bohr radius in MAPbI_3_^23^ so that the wave functions of electrons and holes still overlap, allowing radiative recombination. For large grains, the electron and hole displacements can be much larger, comparable to layer thickness and much larger than the Bohr radius, leading to a large decrease in the charge carrier recombination rate, even at low applied voltages.

**Figure 5:**
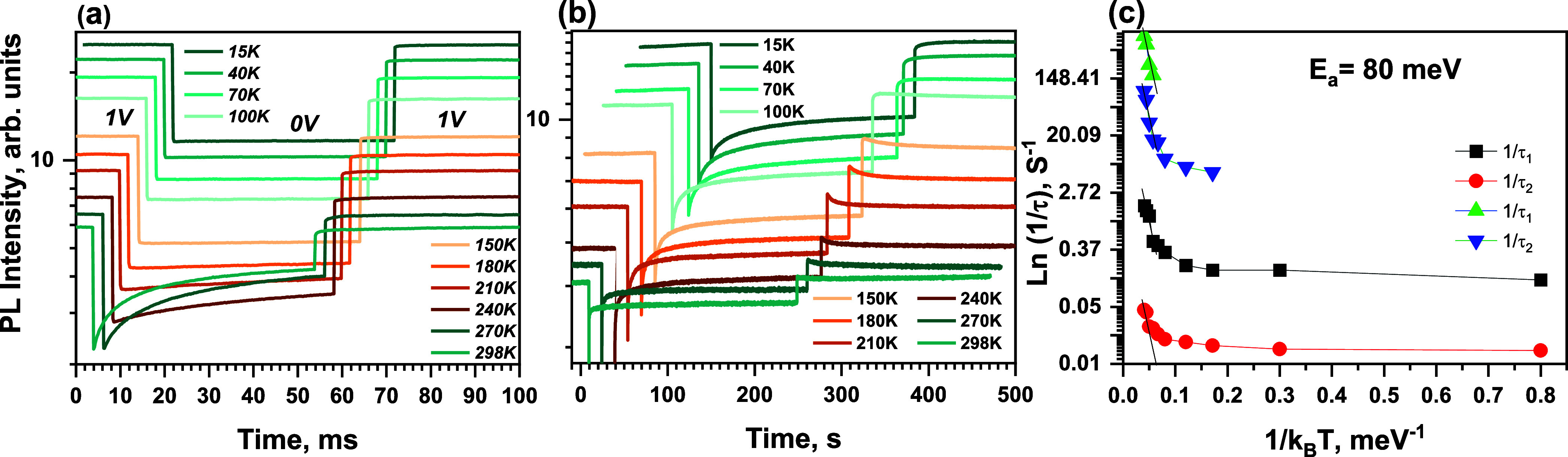
PL intensity kinetics induced by pulsed voltage drop from +1 to 0 to 0 V at different temperatures measured on (a) millisecond and (b) second time scales. (c) Shows an Arrhenius plot for four rate constants obtained from a biexponential approximation of PL recovery kinetics measured on millisecond (green and blue curves) and second (black and red curves) time scales. Black lines show approximations by 80 meV activation energies.

To obtain additional information about the field screening effect, we measured the PL dynamics induced by rectangular voltage pulses at different temperatures. Depending on temperature, the dynamics of PL recovery occur over a wide time range; therefore, we have measured it on a time scale of milliseconds and seconds. Figure 5a shows the PL dynamics obtained at various temperatures under a 50 ms negative voltage pulse from a compensating voltage of 1 to 0 V. The PL intensity under the 1 V voltage increased more than four times when the temperature was reduced to 15 K. Such an increase in PL intensity at low temperatures is a well-known phenomenon.^24,25^ However, the initial relative PL quenching (PL drop normalized to its value at 1 V) is almost independent of temperature: PL intensity drops by ∼2.9 times at 300 K and ∼2.3 times at 15 K.

The PL recovery dynamics attributed to the ion motion slow spectacularly at low temperatures. PL intensity during the 50 ms pulse shows almost no recovery at 180 K and lower temperatures, suggesting that the electric field screening does not occur on a millisecond time scale. However, as Figure 5b shows, it continues on a second time scale. To better characterize the decay kinetics, we have approximated it by a multiexponential function and plotted the obtained rate constants (1/τ) in the Arrhenius plot presented in Figure 5c. The two faster and the two slower rate constants were obtained from measurements in millisecond and second time scales respectively. At high temperatures down to about 200 K, all time constants approximately follow the Arrhenius dependence 1/τ ∼ exp(−*kT*/*E*_a_), with the activation energy of ion motion *E*_a_ ≈ 80 meV being identical to experimental accuracy. Similar low activation energies were theoretically predicted both for iodine vacancies and interstitials.^26^ Slightly larger activation energies of about 137 and 190 meV have also been evaluated experimentally.^9,27^ By investigating EL growth in identical MAPbI_3_ PeLEDs, we have also evaluated activation energies of about 180 meV. On the other hand, the PL recovery at temperatures below 100 K becomes almost independent of temperature. This suggests that either slow ion motion still continues at low temperatures by tunneling or some other temperature-independent or weakly dependent processes cause electric field screening on a minute time scale. For example, electron or hole trap states may be populated, causing the formation of space charges that screen the electric field. The population of shallow trap states in MAPbI_3_ has been observed experimentally at temperatures below ∼100 K,^28^ i.e., approximately when deviation from the Arrhenius law for the field screening rates starts (Figure 5c).

## 3 Conclusions

EFILQ of MAPbI_3_-based PeLEDs was investigated using steady-state, pulsed, and sinusoidally modulated voltages, as well as ultrafast time-resolved PL decay measurements. Two main mechanisms were found to be responsible for the voltage-induced PL efficiency variations: (a) slow irreversible or partially reversible changes of the perovskite layer occurring on a time scale of seconds and minutes and (b) electric field-induced changes in the density and spatial distribution of the photogenerated charge carriers. The extraction of charge carriers from the perovskite layer and the spatial separation of electron and hole “clouds” cause PL changes on a time scale of tens and hundreds of picoseconds. While PL changes within several ps time resolution, we attribute this to the electron and hole displacement within individual perovskite grains. The electronic PL quenching part depends only weakly on temperature when it changes from room temperature to 15 K. However, this part is strongly affected by the electric field screening created by mobile ions. The mobile ions reduce the relative PL quenching several times within tens of milliseconds at room temperature and within tens to hundreds of seconds at temperatures below 100 K. All these processes should be taken into account when analyzing the performance of perovskite devices, as they cause the hysteresis of solar cell operation and the complex EL dynamics of perovskite LEDs.

## 4 Experimental Section

### 4.1 Device Fabrication

Device fabrication was described elsewhere.^14^ Briefly, PolyTPD was spin coated on the precleaned ITO substrates at 4000 rpm for 40 s and then annealed at 150 °C for 20 min, followed by treatment with O_2_ plasma for 6 s at a power of 100 W to improve surface wettability. Afterward, for the small grain device, 0.2 M MAPbI_3_ with 20 mol % extra benzylammonium iodide was deposited on PolyTPD in a N_2_-filled glovebox using an antisolvent method. Then, PCBM solution in chlorobenzene was deposited at 3000 rpm, followed by depositing ZnMgO nanoparticles in ethanol at 4000 rpm. The devices were finished by thermal evaporation of 100 nm Al. The device area is defined by the shadow mask and was 0.125 cm^2^ with dimensions of 2.5 × 5 mm. For the large grain device, 418 mg of FAI, 70.2 mg of CsI, 71.8 mg of PbBr_2_, 54.7 mg of MACl, and 1145 mg of PbI_2_ were dissolved in 2.0 mL of mixed solvents (DMF/NMP = 9:1, volume/volume) to prepare the Cs_0.1_FA_0.9_PbI_2.855_Br_0.145_ perovskite solution. The precursor was diluted to 0.5 M prior to spin-coating. The sample was gas-quenched for 15 s after the start of the spin-coating with a N_2_ gun to form the 150 nm perovskite active layer, similar to the previously reported procedure.^29^

### 4.2 SEM Measurements

SEM measurements of the perovskite gain layer were performed using the FEI Nova 200 scanning electron microscope system.

### 4.3 XRD Measurements

The XRD patterns were recorded on a PANalyticalX’Pert Pro Materials Research Diffractometer using Cu Kα radiation.

### 4.4 Steady State, Alternating, and Pulsed Voltage-Induced PL Quenching and Its Dynamics

Steady-state, alternating, and pulsed voltage-induced PL quenching and its dynamics were measured with the AvaSpec-HS1024 × 58/122 fiber-optic spectrometer (Avantes) or the photomultiplier tube (Hamamatsu H10721-20), depending on the duration of the applied voltage. The sample was biased with a function generator (Tektronix AFG3101), and a continuous-wave 635 nm 5 mW diode laser (CPS635, ThorLabs) was used for sample excitation. A liquid helium cold-finger cryostat (Janis CCS-100/204) was used for low-temperature measurements.

### Ultrafast Time-Resolved PL Investigations

Ultrafast time-resolved PL investigations were performed by a Hamamatsu Streak camera operating in the synchroscan regime. The samples were excited by 515 nm, 80 fs light pulses generated by a femtosecond Yb/KGW laser (Light Conversion Ltd.). The excitation intensity was about 100 nJ/cm^2^. The laser operated at a repetition rate of 80 MHz; however, a mechanical chopper was additionally used to produce pulse packets of ∼500 μs duration at 15 Hz repetition rate. The pulse packets were synchronized with electrical pulses of 25 ms duration. This setup enabled measurements of the PL kinetics at different times before and after the electrical pulse onset by turning the pulse packet arrival time relatively close to the onset of the electrical pulses. The time resolution of the entire system was approximately 13.0 ps.

## Supporting Information Available

SEM images, *J*–*V*–*R*, EQE–*J*, and EFILQ measurement scheme (PDF)
