# Supplementary material for: Electric Field-Induced Quenching of MAPbI3 Photoluminescence in PeLED Architecture
Source: ACS Appl Mater Interfaces. 2023 Aug 30;15(36):42784–91. doi: 10.1021/acsami.3c05880 (PMC11007676; doi:10.1021/acsami.3c05880)
Supplement: Supplementary file 1 — am3c05880_si_001.pdf [file am3c05880_si_001.pdf]

Supporting Information

Electric field-induced quenching of $MAPbI_3$ photoluminescence in PeLED architecture

*Rokas Gegevičius[1], Karim Elkhouly[3,4,†], Marius Franckevičius,[1] Jevgenij Chmeliov[1,2], Iakov Goldberg[3,4],Robert Gehlhaar[4], Weiming Qiu[5], Jan Genoe[3,4], Paul Heremans[3,4] and Vidmantas Gulbinas[1,2]**

[1]Department of Molecular Compound Physics, Center for Physical Sciences and Technology, Saulėtekio Avenue 3, LT-10257 Vilnius, Lithuania

[2]Institute of Chemical Physics, Faculty of Physics, Vilnius University, Saulėtekio Avenue 9, LT-10222 Vilnius, Lithuania

[3]Department of Electrical Engineering, KU Leuven, Kasteelpark Arenberg, 3001 Leuven, Belgium

[4]IMEC, Kapeldreef 75, 3001 Leuven, Belgium

Corresponding Author

*Email: Vidmantas Gulbinas vidmantas.gulbinas@ftmc.lt

(a) (b)

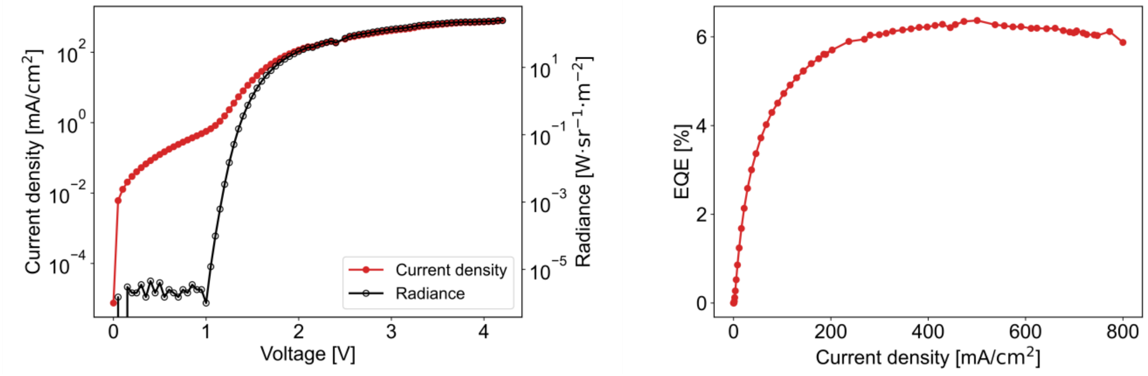

Supplementary Figure S1. (a) Current density- Voltage – Radiance characteristics (*J*-*V*-*R*) of 40 nm $MAPbI_3$ PeLED. (b) External quantum efficiency – Current density (EQE-*J*) curve for the same PeLED.

(a) (b)

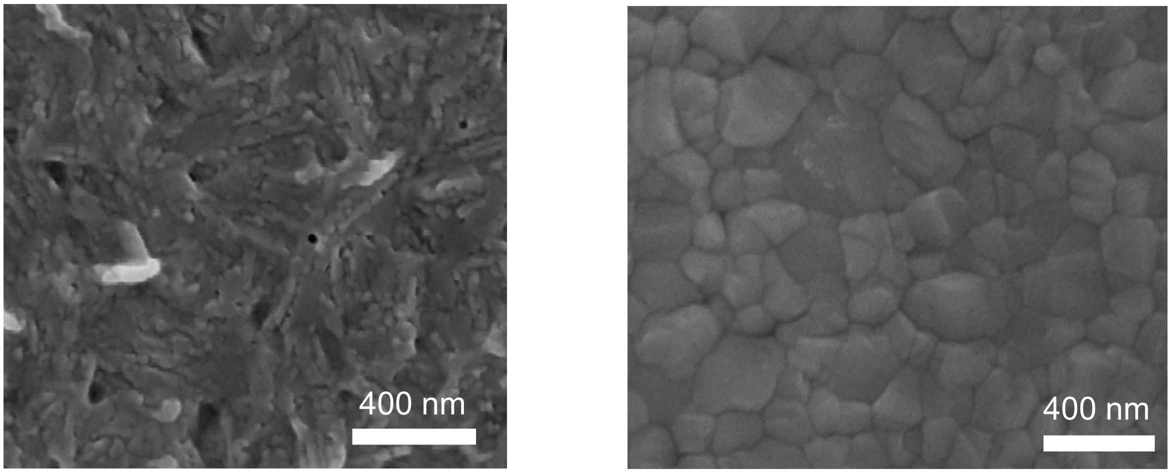

Supplementary Figure S2. SEM Image of (a) small grain 40 nm thick $MAPbI_3$ film on SiO_2/Si substrate; (b) Large Grain 150 nm $Cs_{0.1}FA_{0.9}PbI_{2.855}Br_{0.145}$ film on SiO_2/Si substrate.

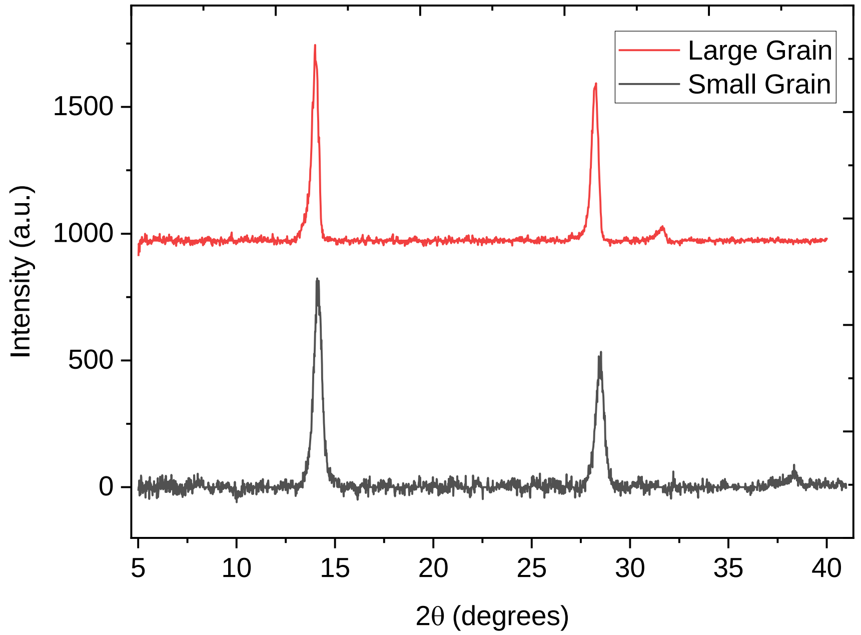

Supplementary Figure S3. XRD peaks for small grain 40 nm thick $MAPbI_3$ film on ITO/Glass substrate, and large grain 150 nm $Cs_{0.1}FA_{0.9}PbI_{2.855}Br_{0.145}$ film on ITO/Glass.

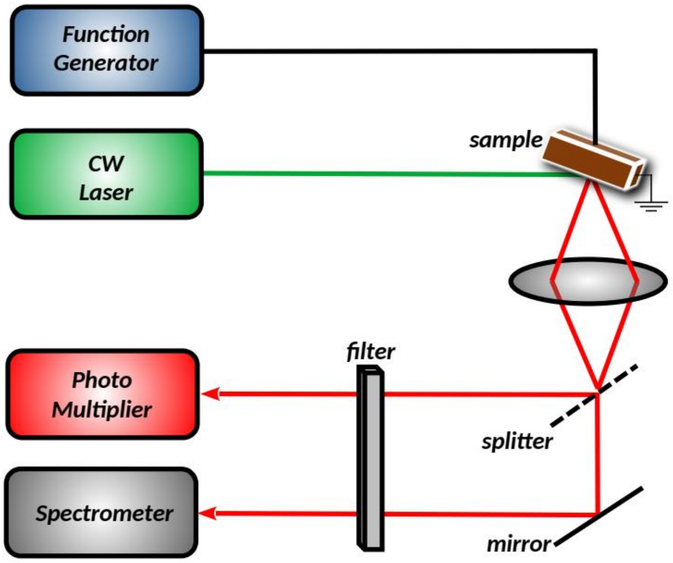

Supplementary Figure S4. Schematic representation of EFILQ measurement scheme in steady state, alternating and pulsed modes.
